# A realist evaluation of the feasibility of a randomised controlled trial of a digital music and movement intervention for older people living in care homes

**DOI:** 10.1186/s12877-023-03794-5

**Published:** 2023-03-06

**Authors:** E.F Ofosu, L De Nys, J Connelly, G.C Ryde, A.C Whittaker

**Affiliations:** 1 Faculty of Health Sciences and Sport, University of Stirling, Stirling, FK4 9LA Scotland UK; 2 School of Cardiovascular and Metabolic Health, University of Glasgow, Glasgow, Scotland UK

**Keywords:** Activity coordinators, Care homes, Digital physical activity, Feasibility, Realist evaluation, Residents

## Abstract

**Background:**

Low physical activity in care home residents brings about negative mental health consequences, such as higher levels of depression and loneliness. With advancements in communication technology, particularly during the COVID-19 pandemic, the feasibility and effectiveness of a randomised controlled trial (RCT) of a digital Physical Activity (PA) resource in care homes deserve more research attention. A realist evaluation was used to uncover influencing factors of a feasibility study implementation to inform how a digital music and movement programme would work and under what circumstances this would be most effective.

**Methods:**

Participants were 49 older adults (aged 65 years +) recruited across ten care homes in Scotland. Surveys were administered at baseline and post-intervention comprising psychometric questionnaires on multidimensional health markers validated in older adults with possible cognitive impairment. The intervention comprised 12 weeks of four prescribed digitally delivered movement (*n* = 3) and music-only (*n* = 1) sessions per week. An activity coordinator delivered these online resources in the care home. Post-intervention focus groups with staff and interviews with a sub-sample of participants were conducted to gain qualitative data on the acceptability of the intervention.

**Results:**

Thirty three care home residents started the intervention, but only 18 residents (84% female) completed both pre- and post-intervention assessments. Activity coordinators (AC) offered 57% of the prescribed sessions, with an average residents’ adherence of 60%. Delivery of the intervention did not go as planned due to Covid restrictions in care homes and delivery challenges, including (1) motivation and engagement, (2) changes in cognitive impairment and disabilities of the participants, (3) death or hospitalisation of the participants and (4) limited staffing and technology resources to deliver the programme as intended.

Despite this, group participation and encouragement of residents supported the delivery and acceptance of the intervention, with ACs and residents reporting improved mood, physical health, job satisfaction and social support. Improvements with large effect sizes were found for anxiety, depression, loneliness, perceived stress and sleep satisfaction, but no changes in fear of falling, domains of general health or appetite.

**Conclusion:**

This realist evaluation suggested that this digitally delivered movement and music intervention is feasible. From the findings, the initial programme theory was refined for future implementation of an RCT in other care homes but future research exploring how to tailor the intervention to those with cognitive impairment and/or lacking capacity to consent is needed.

**Trial registration:**

Retrospectively registered at ClinicalTrials.gov NCT05559203.

**Supplementary Information:**

The online version contains supplementary material available at 10.1186/s12877-023-03794-5.

## Introduction

### Rationale for evaluation

The care home resident population is ageing in the UK [1], following global trends [2], with residents often being diagnosed with more than one long-term health condition (multi-morbidity) [3]. Multi-morbidity increases the likelihood of hospital admission, length of stay and readmission, raising healthcare costs, reducing the quality of life, polypharmacy and mortality [4, 5]. While morbidity consistently predicts care dependence, physical multi-morbidity conveys a lower risk than multi-morbidity with mental and cognitive disorders [6] This is a significant finding, as about 70% of all care home residents have dementia or other cognitive impairments [7]. Further contributing to these issues, the adverse consequences of reduced or low physical activity (PA), or the cessation of exercise, which is common in a care setting, brings about its own negative mental health consequences, such as higher levels of depression [8] and loneliness [9]. Previous research has focused on developing Physical Activity (PA) interventions in care homes to improve the health of care home residents. With advancements in communication technology, particularly during the COVID-19 pandemic, the feasibility and effectiveness of digital PA resources deserve more research attention.

PA interventions may be an excellent means of improving multidimensional health in older adults [10]. This is evidenced by favourable effects on endocrine profiles implicated in human ageing [11, 12]. Further, these have shown improvements in physical health, such as gait speed, balance and performance in activities of daily living [13], as well as in cognitive function [14] and mental well-being [15], such as anxiety [16] and depression [17]. Therefore, considerable effort has been devoted to increasing PA and prescribing exercise programmes in older adults, such as the care home resident population [18]. PA interventions, including multicomponent (chair-based) exercises or dancing, have been shown to improve cognitive function, physical and mental well-being in this population [19–25].

Increasingly, innovative digital resources have been developed to influence PA in care home residents positively. For successful implementation of digital health interventions designed for older adults, understanding the feasibility regarding organisational and systems readiness and the acceptability of digital interventions are fundamental. Recent systematic reviews showed that digital resources to promote PA in older adults were reported as feasible and well accepted [26, 27]. The review by Diener et al. (2022) suggests considering one's physical and cognitive abilities and providing options for individual tailoring of these interventions. It further highlighted that conditions and resources in care homes, such as equipment, physical space, and financial capacities regarding the technology, should be considered [26]. Both reviews [26, 27] emphasised that, although technology-based interventions to promote PA show promising future avenues, more quality studies reporting accurate measures of exercise adherence and effectiveness are warranted.

Further, when designing and testing interventions in the complex situations that care homes reside in, it is crucial to address 'what works, for whom, under what circumstances, and how' [28]. Few studies consider the role of context, mechanisms and external factors (moderators) affecting the effectiveness of a PA programme. In order to expand and translate the roll-out of effective interventions, more information is often needed about how a programme might be replicated in a specific context or whether trial outcomes are reproducible [28]. Therefore, a realist evaluation [29] was chosen for the present study to understand how a digital music and movement intervention might generate different outcomes in different circumstances. Exploring and identifying the how, why, for whom, to what extent, and in what context interventions work could allow researchers and practitioners to understand how to adapt interventions to a new context [30] and further inform decisions about scaling up the programme to deliver to more care homes and influence public health initiatives.

To this end, following the realist evaluation framework, a context (C), Mechanism (M), and outcome (O) configuration [29] was identified to explain the role of the complex situation of UK care homes in which PA programmes are to be rolled out (see Table 1). The effects of a feasibility trial (danceSing Care programme) on multidimensional health outcomes were discussed in light of this CMO configuration. Thus, the two overarching aims of this project were: (1) to determine the feasibility of a future mixed-methods RCT of digital music and movement resources in a care home, and (2) to use a realist evaluation approach to evaluate the process, outcomes and influencing factors of the trial implementation to inform how this programme would work and under what circumstances this would be most effective. With insights into whether and how the outcomes were affected, the initial programme theory will be refined for future implementation in a randomised controlled design in other care home sites.

**Table 1:** Context-Mechanism-Outcome (CMO) hypothesis

Context-Mechanism-Outcome (CMO) hypothesis					
Realist assumption	Focused question	Realist programme theory	Contexts (set of conditions that exist)	Mechanisms (how might these contexts create change)	Outcomes (what changes result from the mechanisms)
1.Collect information about context, mechanism and outcome to evaluate the programme 2.The programme intends to cause a change	1.What information will be needed and could be collected about contexts? Mechanisms? Outcomes? 2.What change (outcome) does the trial intend to create Quantitative: Feasibility, adherence, surveys about psychosocial health markers Qualitative: Interviews and focus groups about acceptability, engagement, delivery and feasibility	1.Identifies intended outcomes 2.Identifies the data necessary to test the programme theory	Situational High burden of the COVID-19 pandemic on the care home system in Scotland Little time and resources for the staff (already happening before COVID-19)	Little time and resources for the staff, understaffing, absence or isolation of staff and participants through restrictions	Complicate readiness for programme delivery and engagement
			Programme 1.Implementation of the danceSing care programme 2.Flexibility from research staff and stakeholders from the danceSing care team	Supporting care home staff, boosting feelings of connection and engagement through group training, meetings and welcoming participants in the ‘danceSing care family’	1.Improve psychosocial health markers of residents 2.Improve programme delivery and engagement

### Programme theory

The programme theories, assumptions, and the logic model underpinning the danceSing care programme are shown in Supplementary Figures S1 and S2.

Programme theories are configured as “context-mechanism-outcome” (CMO) hypotheses in a realist evaluation. This explains how and why different outcomes are generated in different contexts. Linked sets of hypotheses are likely to be produced because different mechanisms will be activated in various contexts, resulting in various outcomes. Generally, hypotheses are developed by asking four questions: (1) For whom will this basic programme theory work and not work, and why? (2) In what contexts will this programme theory work and not work, and why? (3) What are the main mechanisms we expect this programme theory to work? (4) If this programme theory works, what outcomes will we see? The basic programme theory was in line with the aim to test the feasibility of an RCT implementing the danceSing care programme in specific UK care homes, and measuring psychosocial health markers. This is reflected by the stated realist assumptions (Table 1).

This programme theory could be further subdivided into the following theories:

1. If the danceSing care resources are delivered to the care homes, the Activity Coordinators (ACs) would consistently deliver the programme to the residents.
2. If the programme is delivered in the care homes, the residents would want to regularly take part.
3. If the ACs provide the programme consistently, the participants would experience improved psychosocial health markers. Changes in pre-and post-survey data and qualitative interviews would evidence this.
4. If the ACs are given enough organisational support such as resources and time, they would be engaged in this programme. This would therefore, establish shared learning and co-production between programme developers, care homes, and researchers.
5. If the group sessions are adequately and consistently used, residents would feel more engaged in group activities, creating a community. This communal feeling would increase self-confidence and quality of life and reduce loneliness. This could potentially inspire future usage of the programme's resources.

Further, researchers, stakeholders from the to-be-tested programme, and representatives from the UK care homes where the programme would be implemented developed the focused questions and considered the set of conditions that existed (Context), how these contexts might create change (Mechanism), and what changes would result from the mechanisms (Outcomes). The study’s hypotheses were developed based on the assumptions and the focused questions. Therefore it was necessary to collect information about context, mechanism and outcome and identify features of implementation or organisation that affect whether or not the trial works [31]. The different CMOs are listed in Table 1. Each row in this table represents the outcome produced by a particular mechanism in a particular context. This was followed by retroduction, a form of logical inference using abductive reasoning to identify the most likely explanations for an observed data set. This retroduction was done by interpreting and analysing the data applying the configured CMO. Finally, these interpretations served as means to make refinements to the initial programme theory for future implementation, as outlined in the discussion section.

### Specific research objectives

In line with the overarching aims of this study outlined above, specific research objectives were to evaluate the following topics: (1) Feasibility: was the activity implemented and/or delivered as planned? Were adherence rates at an acceptable level? Were the outcomes adequate and realistic for this programme and setting? (2) Context: What were the potential barriers for care homes or ACs to provide these resources? Were the programme’s resources suitable for this setting? (3) Mechanism: what underlying mechanisms made the danceSing Care programme work (or not)? Was it the situational context or the programme context? (4) Outcome: What changes resulted from the mechanisms?

## Methods

### Design

This study combined two evaluation approaches: first, a mixed methods feasibility approach and a realist evaluation approach. Initially, a mixed-methods research approach to a 12-week feasibility trial in care homes with pre- and post-intervention collection of quantitative and qualitative data about feasibility (adherence, safety, adverse events) and participants' multidimensional health. This was to determine (1) an appropriate way to deliver the programme in this setting and (2) the appropriate secondary outcome measures for a future RCT. This was preceded by implementing an expert advisory group (AG). The AG consisted of danceSing Care staff, the research team, representatives from care homes and older adult members of the 'Stirling 1000 Elders' (https://1000elders.stir.ac.uk/, a group of adults aged 60 + who have signed up to participate and engage in research on older adults at the University of Stirling). The outcomes were reported following appropriate CONSORT guidance for feasibility trials [32]. The study was approved by the University of Stirling NHS, Invasive and Clinical Research ethics panel, project NICR 3735 and retrospectively registered at ClinicalTrials.gov, NCT05559203 on 29/09/2022.

Second, the evaluation questions and scope evolved beyond initial questions regarding feasibility throughout the evaluation because the capability of delivering the programme and engaging the residents by the activity coordinators was lower than expected. To uncover the underlying factors that explained the implemented situation (such as relevant context (C), mechanisms (M), and outcomes (O)), we additionally used the realist evaluation methodology based on RAMESES II reporting standards [31]. Given the difficulties with delivery, any attempts to establish linear, causal relationships between inputs and outcomes (e.g., by comparing 'pre' and 'post' data) would have been largely meaningless. Thus, using realist evaluation, the CMO configuration was identified to interpret the outcomes according to what happened. Pre- to post-intervention changes in secondary outcome measures were calculated to indicate effect sizes, implement the measures, and test their acceptability before integrating into a larger RCT.

#### Environment surrounding the evaluation

The environment for the evaluation was across ten care homes in Scotland, United Kingdom. All care home operation managers/activity coordinators were provided with the danceSing Care digital resources login details to access weekly pre-recorded movement and music sessions. It was confirmed that all care homes had sufficient technological support to participate in this programme. Before starting the intervention, an in-person training session was organised for care home staff (activity coordinators and operation managers). This training covered general instructions on rolling out the program and addressing any staff concerns about the study, including any IT issues. In addition, a concise information pack was distributed to activity coordinators, including the programme's and researchers' contact details for any questions or problem-solving. All study information was co-designed with the advisory group, who met four times to determine the amount and timing of programme delivery and priorities for and suitability of outcome measures.

### Participant recruitment process and sampling strategy

Participants were recruited across ten care homes in the Balhousie Care group in (https://balhousiecare.co.uk/) between December 2021 and January 2022. Inclusion criteria were (1) residents in care homes ≥ 60 years, (2) able to complete 12 weeks of a movement and music program (3) having the capacity to give informed consent. Participants were not eligible if they (1) were currently taking part in any other clinical trial which could potentially have an impact upon or influence the findings of the current study, (2) had pre-existing conditions or concurrent diagnoses which would profoundly impact their capacity to undergo the intervention, even once adaptations have been made (3) inability to understand written/spoken English adequately to participate in the measures and intervention (e.g., due to cognitive or sensory impairment). Care staff were asked to gauge the interest of residents in the programme and were provided with recruitment flyers. Care staff informally screened interested participants for eligibility with a simplified version of the information sheet. Following this, the research team contacted the respective care homes, provided full participant information sheets, and obtained informed written online consent. Where this was not feasible online, care staff ensured the residents consented to the study on paper copies of the consent form provided at the start of the survey pack. In addition, in-person visits to the care homes were planned if COVID-19 restrictions allowed.

### Programme

The programme was a digital movement and music programme with resources from danceSing Care (https://dancesingcare.uk/). It consisted of three movement sessions and one music session each week, the recommended dose agreed upon between danceSing Care and the Advisory group, each lasting about 20 min. Also, the danceSing care resources were designed to suit older adults with physical and cognitive impairments (residents with mobility aids and/or dementia). Movement sessions included chair and standing fitness, which started with a warm-up and finished with stretching exercises (see Supplementary Table 3 for intervention description). Sessions were managed and supervised by care home activity coordinators.

### Measures

#### Feasibility: adherence and safety

Care home staff were asked to provide the danceSing care physical activity resources three times per week plus one music session. Participants' adherence was assessed by asking homes to record how many sessions they offered each week and the participants' attendance at each of these using a provided register sheet (Supplementary Table S4). This was then calculated as adherence for participants by computing the percentage attended out of the number of sessions offered by the care home. Care home adherence was calculated as the percentage of offered sessions from the recommended 3 + 1 × 12 weeks. Adherence data were requested weekly via a reminder email with a specific register provided to each care home. This was to indicate attendance at the sessions provided per participant and any additional attendance from non-participants. Researchers logged reasons for withdrawal weekly. Adherence data missing was followed up with phone calls to each home from the research team to help assess any difficulties in delivering the programme. Researchers kept a reflective log of any additional information gathered in this way.

As part of the care home staff workshop before the intervention, activity coordinators were advised to ensure residents engaged in the danceSing care programme according to their capabilities. Adverse events related and unrelated to the intervention were reported weekly by activity coordinators via email or telephone in addition to the adherence reports.

#### Socio-demographics and multidimensional health

The pre-and post-intervention survey recorded basic demographics such as age, gender, relationship status, ethnic group and level of education. Postcodes were not included to compare against the Scottish Index of Deprivation because participants were residents of care homes.

After discussion with the AG, a list of priority and appropriate outcomes for limited efficacy testing in this feasibility study was devised. The key areas included: the number of falls in the past three months, activities of daily living and health, psychosocial well-being (anxiety, depression, stress and loneliness), sleep satisfaction and frailty measures such as physical function, appetite and weight loss. These multidimensional health markers were assessed using standardised questionnaires validated in older adults with mild cognitive impairment. The internal consistency of the measures are reported as the Cronbach’s alpha with values ≥ 0.70 indicating good levels of reliability.

Fear of falling was measured using the Falls Efficacy Scale – International (short form) (FES-I) [33]. It is a seven-item scale that measured how concerned participants were about falling during social and physical activities on a Likert scale ranging from one (not at all concerned) to four (very much concerned). It has been validated in older adults with a Cronbach's alpha of 0.92. Total scores ranged from seven to 28, with higher scores indicating severe concern [33].

Two measures were used for daily living and health-related quality of life. The Dartmouth Cooperative (COOP) Functional Assessment charts measured participants' physical fitness domains, feelings, daily and social activities, changes in health and overall health using pictures [34]. The European Quality of Life 5 Dimensions 3 Level Version (EQ-5D-3L) [35] evaluated five dimensions of health (mobility, self-care, usual activities, pain/discomfort and anxiety/depression) with responses ranging from no problems to considerable problems on a 3-point scale, and finally a rating of general health from 0 o 100.

Measures of anxiety, depression, stress and loneliness were used to assess psychosocial well-being. Anxiety and depression were measured using the Hospital Anxiety and Depression Scale (HADS) [36], a 14-item questionnaire scored on a four-point scale that measures anxiety and depression on two subscales. Each subscale of the HADS has seven items giving a maximum score of 21, with scores ranging from zero to seven considered as normal, eight to 10 as borderline and 11 or more as a significant case of anxiety or depression [36]. The Perceived Stress Scale (PSS) [37] measures how participants perceive life situations as stressful. It has ten items with responses ranging from zero (never) to four (very often) and total scores of 0 to 13, 14 to 26 and 27 to 40 depicting low, moderate and high perceived stress, respectively [37]. Also, the brief UCLA loneliness scale (ULS-6) [38] was used to assess subjective feelings of loneliness on a four-point scale (1-never to 4-often) with six items; the greater the ULS-6 score, the more significant the loneliness. Each of these measures has reported Cronbach’s alpha for reliability of  > 0.7 [37–39].

Sleep satisfaction was measured using the National Sleep Foundation's Sleep Satisfaction Tool (SST) [40]. It is a nine-item scale with a Cronbach's alpha of 0.87 and a high score suggesting greater sleep satisfaction [40]. In addition to the SST, an unstandardised item measured how often participants experience disturbed sleep due to noise outside the room. Participants chose responses from options: "not during the past month", "less than a week", "once or twice a week" and "three or more times a week".

Appetite was assessed using the Simplified Nutritional Appetite Questionnaire (SNAQ) [41], which has a maximum score of 20, where a score less than 14 specifies poor appetite and a Cronbach’s alpha coefficient of 0.7.

Participants' weight loss was measured on the weight loss item from the Fried Frailty Scale [42]. Regarding frailty measures, it was set out to assess physical function through the Short Performance Battery [43], complemented with handgrip strength and Timed Up & Go [44] test to indicate frailty. However, due to the COVID-19 restrictions, it was not possible to visit care homes and undertake these assessments.

### Procedure

Surveys were completed at baseline and then again in the four weeks following the completion of the intervention. These were either completed online by residents with the help of the researchers via an online Microsoft Teams meeting or were administered in-person by the care home staff using paper versions. All completed surveys were uploaded onto JISC online survey software (https://www.jisc.ac.uk/online-surveys).

Two focus groups were conducted online in the four weeks following the completion of the intervention lasting on average 55 min each. Collectively, five activity coordinators participated in both focus groups carried out on Microsoft Teams and were recorded following informed consent from the care home staff and research team. Four residents from two care homes consented to be interviewed in person. Due to the changes in cognitive impairment of some residents, recruitment for interviews was streamlined to specific care homes and limited to residents with no or mild cognitive impairment. Data from interviews were audio-recorded on a password-secured voice recorder and transcribed verbatim. Semi-structured interviews lasted approximately 12 min each. The semi-structured interview and focus group questions covered general participation, adherence, programme outcome and programme delivery. These questions were devised by the research team and trialled and altered with the advisory group (Supplementary Material S5 for the interview and focus group guide).

#### CMO configuration

The CMO factors were generated via monitoring the care home environment. Weekly phone calls and emails provided qualitative and quantitative data about the ongoing trial adherence and situation in the care homes. The interviews and focus groups conducted post-intervention were adapted according to realist evaluation strategies [45].

### Data analysis

Quantitative data, such as primary outcomes of adherence rate, or the secondary survey outcomes, were analysed using the IBM SPSS Statistics version 26.0. The means, mean differences and 95% confidence intervals (CI) were reported for continuous data, and a count (number, %) was reported for nominal data. Pre- to post-intervention scores for secondary outcomes were compared using the paired sample t-test, and Cohen's d was calculated as a measure of effect size and was interpreted as small (d = 0.2), medium (d = 0.5), and large (d = 0.8) [46]. All testing was two-sided with a significance threshold of < 0.05. Qualitative data from interview and focus group transcripts were analysed using NVivo (released in March 2020).

**Fig. 1:**
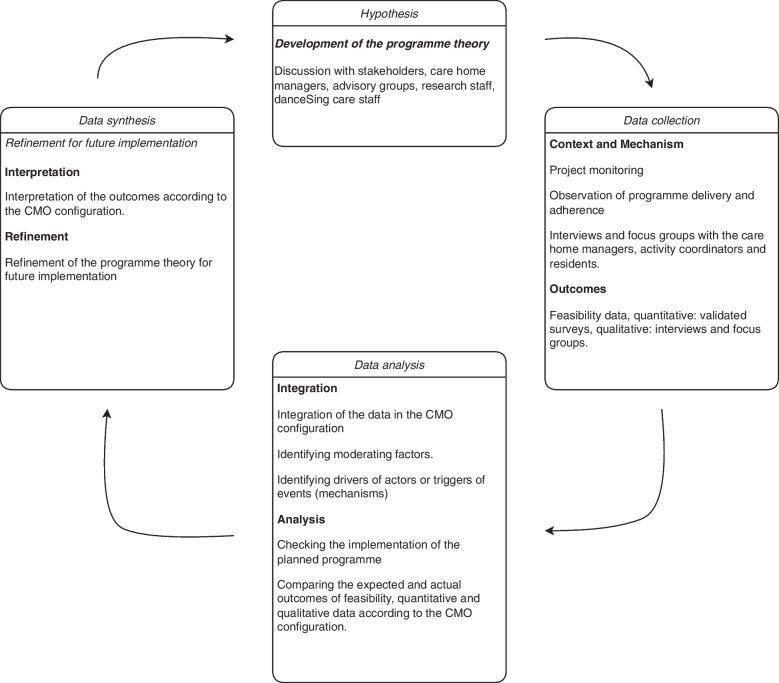
Process of the realist evaluation

The research team then integrated and evaluated the outcomes of the feasibility trial according to the identified CMO configuration. Recurring resources or contexts in which the programme took place that the residents or activity coordinators described as helpful or influential (or not) were considered as a Context (C) or Mechanism (M). These descriptors generated the overarching themes of the CMO configuration. The researchers then used these interactions as contextual narratives to identify why residents responded as they did. Accordingly, the research team, the AG, and the stakeholders discussed interpretations of actions or events. These interpretations were then tested by seeking data to either support or contradict the statements (either via data on current events or from the interviews and focus groups). Finally, these interpretations were considered when refining the programme theory for future implementation. The discussion section elaborates further on these refinements. Figure 1 shows the process of the realist evaluation.

## Results

The following section consists of five sub-sections, including details of participants, the feasibility of the intervention, delivery challenges, intervention effects and refinement of the programme theory for future implementation. The qualitative and quantitative analysis findings are presented together in each section where data from both were available.

### Participants

**Fig. 2:**
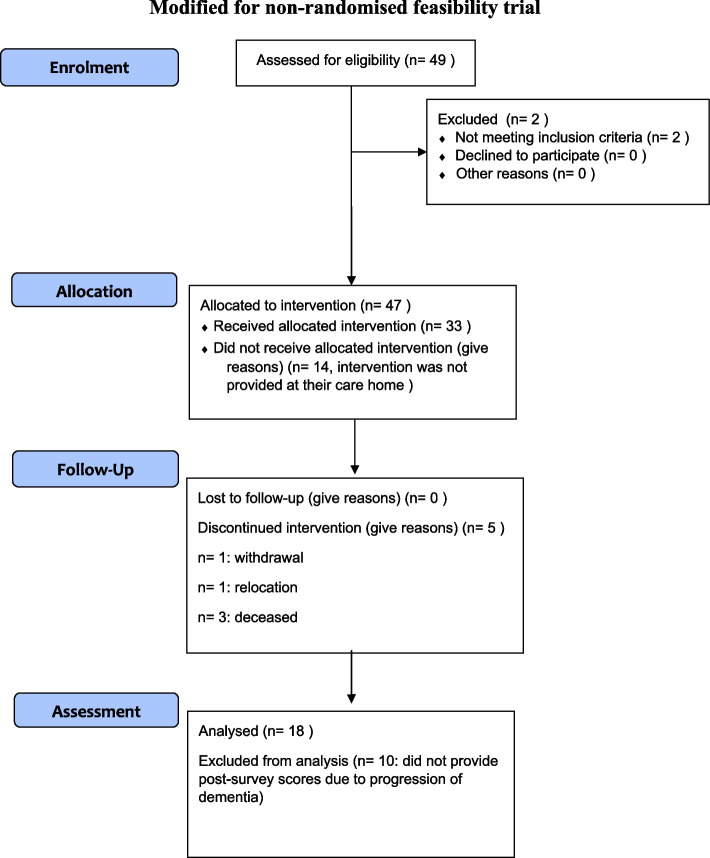
CONSORT diagram

Of the 49 participants approached for recruitment, 47 were enrolled in the study and completed baseline measures, and 33 residents took part in the intervention; a Consort diagram of the progress through the study is shown in Fig. 2.

Most participants were females (84.8%) of White British ethnicity, with 84.9% aged 75 years and over. Most participants were selected from care home units for older adults with dementia. The four residents interviewed were between the ages of 75 and 90, and three were females. Baseline participant characteristics are detailed in Table 2.

**Table 2:** Participant characteristics

**Characteristic**	**Description**	N(%)	
		*N* = 33	*N* = 18
Age group	60–74 years	5 (15.15)	2 (11.11)
	75–84 years	13 (39.39)	8 (44.44)
	85 or over	15 (45.45)	8 (44.44)
Sex	Female	28 (84.8)	15 (83.8)
Ethnic origin	White—British, Irish, other White	32 (96.97)	17 (94.44)
	Prefer not to say	1 (3.03)	1 (5.56)
Relationship	Single, divorced, widowed	27 (81.82)	16 (88.89)
status	In a relationship/married but living apart	2 (6.06)	1 (5.56)
	In a relationship/married and cohabiting	2 (6.06)	1 (5.56)
	Prefer not to say	2 (6.06)	0 (0)
Highest level	No qualifications	11 (33.33)	8 (44.44)
of education	Did not complete National 5 s/Standard Grades/GCSE/CSE/O-levels or equivalent	3 (9.09)	1 (5.56)
	Completed National 5 s/Standard Grades/GCSE/CSE/O-levels or equivalent (at school till aged 16)	6 (18.18)	4 (22.22)
	Highers/Advanced Highers/AS-levels/A-levels or equivalent (at school till aged 18)	6 (18.18)	2 (11.11)
	Undergraduate degree or professional qualification	1 (3.03)	1 (5.56)
	Prefer not to say	6 (18.18)	2 (11.11)

### Feasibility of the intervention

Regarding feasibility, 47 participants from 10 care homes fulfilled the inclusion criteria, consented, and completed the pre-intervention surveys. Seven of these care homes provided the intervention programme, enrolling 33 residents in the intervention. Five dropouts were noted: one due to withdrawal, one relocation and three residents who passed away during the intervention. Care homes delivered 57% of the sessions (193 of 336) over the 12-week intervention period, with an average residents' adherence of 60%. Quantitative adherence statistics are shown in Table 3. Finally, 18 (54.55%) residents had sufficient cognitive ability at the follow-up to provide meaningful post-survey scores as informally assessed by the researchers with assistance from care home staff. Where it became clear that participants had declined in their cognitive ability at follow-up beyond the ability to take part, the post-survey was immediately terminated by the researchers as it was not deemed ethical or meaningful to continue.

**Table 3:** Adherence data

Number Sessions Offered															
**Care Home**	**Wk 1**	**Wk 2**	**Wk 3**	**Wk 4**	**Wk 5**	**Wk 6**	**Wk 7**	**Wk 8**	**Wk 9**	**Wk 10**	**Wk 11**	**Wk 12**	**Total**	**% Sessions Offered**	**Average sessions per week (median: 3)**
** CH1**	4	4	4	4	0	0	4	4	4	4	4	4	40	83.33%	3
** CH2**	4	4	0	0	0	0	0	0	0	0	0	0	8	16.67%	1
** CH3**	4	1	2	0	4	3	0	1	3	3	4	3	28	58.33%	2
** CH4**	1	4	3	3	1	3	4	3	4	3	3	4	36	75.00%	3
** CH8**	2	2	2	0	1	0	0	0	0	0	0	0	7	14.58%	1
** CH9**	4	4	4	0	0	4	2	4	0	0	4	4	30	62.50%	3
** CH10**	4	4	4	4	4	4	2	4	4	3	3	4	44	91.67%	4
	**23**	**23**	**19**	**11**	**10**	**14**	**12**	**16**	**15**	**13**	**18**	**19**	**193**	**57.44%**	

Note: *CH*-Care home. Of the 10 care homes that we identified to start the programme, seven started and completed the 12-week programme

The interviews and focus group data derived five central themes with sub-themes on feasibility. For data integration and analysis, themes on feasibility were linked to the CMO configurations, which revealed sub-themes such as adherence, delivery challenges, modifications and intervention effects, as summarised in Table 4.

**Table 4:** Data integration and analysis

	**Feasibility**	**Context**	**Mechanism**	**Outcome**
Specific research objectives	Was the activity implemented and/or delivered as planned? Were adherence rates at an acceptable level? Were the outcomes adequate and realistic for this programme and setting?	What were the potential barriers for care homes or ACs to provide the resources? Were the programme’s resources suitable for this setting?	What underlying mechanisms made the programme work (or not)? Was it the situational context or the programme context?	What changes resulted from this?
Themes derived from the data integration and analysis	-Adherence	-Delivery challenges -Facilitation resources -Cognitive impairment	-Motivation and engagement -Social support -Job satisfaction	-Qualitative and quantitative data about psychosocial health markers of participants

### Adherence

Participation and class size were sub-themes from the central theme of adherence. Care home staff described residents' participation in sessions as 'not as expected' due to several delivery challenges. For example, one care home staff member that struggled to get residents to adhere to the recommended dose of the intervention mentioned:

"We (care home) did really struggle, especially at the start, and once the COVID cleared, it got a little bit better for a couple of weeks, and then it slipped back into them not really engaging in it."

However, overall, the intervention was accepted by residents and this was seen in their participation and records of adherence. The digital intervention was an activity that the residents looked forward to. They referred to it as meaningful and purposeful. Some care home staff made comments like:

"Most of our residents participated in all of the sessions. There were a couple of residents that missed a few; they just did not feel like it that day, or they were out with their families and things like that, but all in all, it was a good turnout."

and:

"They (residents) started to look forward to it. You know, they remembered it. It was really great. It was excellent."

Also, in terms of participation, the number of sessions offered to residents varied across care homes. Some care homes offered more sessions than the recommended dose, whereas others offered fewer. In the focus group discussions, care home staff stated in response to them being able to engage residents in three movement and one music session a week:

"I (AC) think we (care home) have done really well. The attendance was quite good. Actually, more residents participated, and we weren't doing this like three times a week, but probably four or five. I think it went great."

but also:

"…I (AC) did probably, I have run it once per week, or maybe twice per week, sometimes in different parts of the care home."

Class size differed in the various care homes, with some care homes recording an increase in the number of residents participating in the intervention and others seeing a decrease, again due to the challenges of delivering an intervention specifically in a care home setting. Care home staff explained that residents were lost to death, dementia and COVID-19 in comments such as:

"Two residents passed away, but we got a new resident, and then it was right up her street like music, dancing and exercise."

and:

"Decrease for me (AC), but again it was like I said, the three residents in particular that their level of dementia got worse, they became unwell and just could not continue with the programme"

and:

"I (AC) think I had the decrease just because we had COVID in the last month, just at the end of the 12 weeks. So that is why I could not really do any dancing and singing with people."

### Delivery challenges with the intervention

As mentioned above, difficulties with engagement and delivery were noted early on, and the CMO evaluation was applied. Difficulties noted in the researchers' logs implied a complex underlying situation in care homes vastly affected by e.g., the COVID-19 pandemic, wider policy environment and understaffing (Context). This could affect motivation and engagement to either deliver the programme or attend a session (Mechanism), affecting adherence rates and possibly implicating the psychosocial outcome measures. More details of these emerged in the qualitative adherence data below. However, Supplementary Table S6 summarises the challenges that came with the delivery of the intervention.

#### Motivation and engagement

One integral part of the feasibility outcomes was delivery challenges which influenced adherence and informed the future modifications for the intervention discussed by the care home staff. The main challenge reported by care home staff was that participants were less motivated to engage in the programme, primarily at the initial stages. Also, participants initially keen to engage in the programme were demotivated when other participants withdrew or refused to participate. Participants also voiced that they were not enthused to participate all the time. In addition, residents' unavailability due to external visits to the care homes was a challenge. ACs and participants expressed these challenges by saying:

"The main difficulty was motivation, but that is really just at the start because once the residents are down and we begin, they are so happy, keen really focused on the exercise and things. So it was a small starting issue, but it did not last very long."

and:

"Some weeks you are in the mood and other weeks you are not. You get in a mood. You know I (resident) am 76 now and some days you just cannot be bothered."

and:

"There were a couple of residents that missed a few; they just didn't feel like it that day, or they were out with their families."

and:

"There was one resident who enjoys exercises every day, she was always keen and motivated, but when it just came down to her participating alone, that was when she stopped. Nobody else was wanting to do it along with her, so she felt that she was on her own. She got less motivated to do it."

#### Health conditions

Further, cognitive impairment and disabilities of the older adults was a potential barrier for ACs to provide the resources. One of the concerns raised by care home staff was the challenge of working with older adults with significant cognitive impairment and disabilities. Residents with dementia sometimes looked confused during sessions. Other residents with physical disabilities, such as mobility and hearing problems, did not participate fully in singing or movement sessions. ACs said:

"There were three residents in particular that their level of dementia got worse, they became unwell and just could not continue with the programme"

and:

"Most residents have got difficulties with mobility, so some of the exercises were not very suitable."

and:

"Like I (AC) said, the singing sessions did not go down well with people. They (residents) really didn't participate fully, for example, one resident has hearing problems and could not sing along.

#### Death and hospitalisation

ACs explained that some participants were lost to death or admitted to the hospital and were unable to continue participation:

"There was one resident who went to hospital and was admitted to hospital. She was not able participate anymore after that."

and:

"We (care home) had two residents who passed away. Also, one was in the hospital for a few weeks as well."

Moreover, the closure of care homes due to the COVID-19 outbreak disrupted participation in the programme as residents and care home staff were isolated for weeks. For example, an AC said:

"Our home was closed for about four weeks with COVID, so we had residents at all times isolate in their bedrooms. As we were all in bubbles, we did try one to one sessions, but obviously we would have to be in PPE and if they had COVID, they were not feeling up to. It was about four- or five-weeks chunk that we were not able really to participate much at all in the programme".

#### Resources for delivery

ACs highlighted a lack of resources for delivery, including staff shortage and absence, technical problems and limited space for running sessions. As part of the discussions, it was recorded that there was a high staff turnover across care homes. ACs had no replacement care home staff to take up their duties when they were on holidays or isolated due to COVID-19 during the 12 weeks of the intervention. Also, ACs did not get any support from other care home staff. This was due to either the heavy workload on care home staff or a shortage of care home staff on duty. It was also reported by some care homes that poor internet connection and faulty display screens affected getting access to and viewing the digital intervention. Again, ACs expressed their struggles to set up lounges for sessions as the space is also shared with other residents not included in the study. In line with the sub-theme, 'facilitation resources' ACs put this across by saying:

"The internet over here (care home) is not great because we live in the countryside. Our signal is poor, so we have problems with buffering, loading and refreshing the page".

and:

"We (care home) did have a technical hitch for about 3 weeks because we could not get the programme displayed on the TV's. Once the hitch was over, it was easy enough for the programme to go on our screens"

and:

"…but it could have to do with our (care home) setting because they (residents) were in a lounge with other residents that were not participating. It was purely the fact we were short staffed, and the carers were not always there to transfer them into another room to do it."

and:

"We (care home) found it difficult at times and just purely from separating the participants into another room due to staff shortages. We did come up with trouble of staffing."

#### Progressive and future modifications

During the 12-week intervention, some changes were made by ACs to help residents adapt well to the intervention. Progressive modifications that care home staff found helpful in facilitating the programme included making participation a part of the care home schedule with an allocated time slot, playing music at the start of sessions, and briefing and encouraging residents before the start of sessions. These were expressed as:

"Well, I (AC) adopted an early pattern, about the 11:30 mark in the morning once everybody was up. They would have had their morning snack, the coffee and that time before lunchtime was always a good time. Before lunchtime is when I (AC) like to get the group together for activities. Whether crafts, music or live entertainment, it was a good period to get people together".

and:

"We (care home) would always put the music on first, to lift their moods and try to get them to engage more in the exercises."

and:

"We (care home) definitely found telling them in advance helped, so that they knew it (the intervention) was going to be happening the next day. Also, like I (AC) said, we did the music part first before the exercises to try and lift their mood and hope that more residents would take part."

and:

"I (AC) spend lots of time trying to encourage people. I had to go and speak to them (residents)."

Additionally, focus group discussions pointed to other alterations and actions that could be made to enhance the feasibility and future implementation of the intervention in care homes, leading to future modifications. It was suggested that the number of sessions be reduced from four sessions a week to two or three sessions a week to recruit residents with low or no cognitive impairment and to play background music during movement sessions. Comments on future modifications included:

"Yeah, I (AC) would run the programme again, but not for four times per week. It is a little bit too much and you have to do other activities as well, not just sing and dance all the time. I will probably be doing it again once or twice per week. I think it is more than enough."

and:

"I (AC) have noticed the music work. It is always better when they can hear songs they are familiar with. I think it would be better if there was louder music at the back in the background."

and:

"I (AC) definitely we would maybe choose a different group of people now because we have had different residents come in that I feel would probably be more suitable for it now than the initial ones we started with but certainly we would do it (intervention) again".

### Intervention effects – Focus groups

From the qualitative data, perceived intervention effects were reported by both residents and care home staff. Sub-themes were improved mood and physical health, care staff job satisfaction and social support.

#### Mood and physical health

It was mainly stated that the intervention improved the mood of residents. In some cases, an increase in residents' physical capabilities was seen:

"…and then as soon as the session starts their mood just goes up and up, and by the end of it (intervention session), they (residents) are so happy. We have got one lady who quite often would stand up and shake her hips and she was very motivated and excited. So, it was really great."

and:

"It (intervention) definitely improves moods. The difference between the start of the session and the end of the session with their moods was quite a big change."

and:

"Probably for one resident, I (AC) would say as the sessions went on, she done a little not much, but a little bit more each one so I do not know if that's been physical for her meaning it has made her physically a bit stronger."

#### Job satisfaction

Care home staff also acknowledged that facilitating the programme gave recognition to them in the care home, gave them a sense of fulfilment and added to their knowledge of physical activity. Some excerpts affirming the importance of the intervention to care home staff were:

"I think it made us feel good to see them happy and made you think that that you have done a good job. So it is just a feel good thing and makes you go home at night and think I have had a good day."

and:

"I have gained loads through this experience, so I have learnt new movements which are safe and easy to follow. It was great to do some stretches in the morning as well for everyone and sometimes other staff would join and enjoy it too."

and:

"It has been very beneficial for me. I was able to get to know people (residents) better as well and they (residents) recognise me now. They do recognise me and sometimes they would just follow me. Some residents would just follow me because they know there will be something fun happening.”

and:

"They look at us (ACs) different from the carers because we have the time to spend with them and have fun with them."

#### Social support

Residents were happy to work with ACs, built friendships and loved participating in groups. Also, care home staff pointed out that engaging in the programme helped to establish positive resident-resident and resident-staff relationships. Some comments on social support included:

"I (AC) think just like the social kind of bonding, it has brought us (staff and residents) all a bit closer I think."

and:

"They (residents) get really engaged and I think we (care home) have really enjoyed the social part of it as well. Just being with friends and doing something meaningful was great"

and:

"When there is a song playing, the carers around also join in and sing a couple of songs with residents and encourage them (residents) to do a couple of movements."

### Intervention effects – Survey

Pre- and post-intervention data from the surveys provided insights into the multidimensional health outcomes of the participants. Significant changes were noted for anxiety and depression (HADS), loneliness (brief UCLA loneliness scale), perceived stress (PSS), and sleep quality (STT). However, given the feasibility nature of this study and the small sample size, more importance should be given to the mean differences and 95% CI and effect sizes (Cohen's d) presented in Table 5 to show that the intervention yielded positive effects across many measures.

**Table 5:** Effect of intervention on well-being outcomes

Variables	Baseline mean	Post-intervention mean	Mean diff	95% CI	*p*-value	Effect size (Cohen’s d)
FES	8.17	8.72	-.556	[-4.199, 3.088]	.752	-.076
Dartmouth COOP	16.33	15.17	1.167	[-.664, 2.998]	.197	.317
HADS-Anx	6.22	4.11	2.111	[.373, 3.850]	.020*	.604
HADS-Dep	6.17	3.17	3.000	[1.303, 4.697]	.002*	.879
EQ-5D-3L	8.00	7.72	.278	[-.714, 1.270]	.562	.139
Brief UCLA loneliness scale	10.61	8.94	1.667	[.067, 3.267]	.042*	.518
PSS	14.72	10.56	4.167	[.318, 8.016]	.036*	.538
STT	27.06	33.72	-6.667	[-9.174, -4.159]	< .001*	-1.322
SNAQ4	16.39	16.22	.167	[-1.078, 1.411]	.781	.067

Note: *FES* Falls Efficacy Scale International (7-item), *Dartmouth COOP* Dartmouth Cooperative Functional Assessment Charts, *HADS* Hospital Anxiety and Depression Scale, *EQ-5D-3L* EuroQol five-dimensional descriptive system (three level version), *UCLA* University of California, Los Angeles loneliness scale, *PSS* Perceived Stress Scale, *STT* National Sleep Foundation Sleep Satisfaction Tool, *SNAQ4* Simplified Nutritional Appetite Questionnaire

^*^marks significance *p* < 0.05

### Refinement of the programme theory for future implementation

The primary programme theories underpinning the danceSing care programme were described in the Introduction. The outcomes of this study were interpreted according to the CMO configuration as outlined in the process of the realist evaluation (Fig. 1). This revealed practical implications for future intervention in this particular care home setting (see Table 6). The four main refinements towards a future intervention were: (1) implementing two or three sessions/week, (2) encouraging participation in the care homes, (3) adding questionnaires of multidimensional health markers complemented with objective measures in a possible future RCT, (4) considering dementia in the care home population. Consequently, refinements to the initial programme theories are offered to aid the future implementation of this digital music and movement resource in wider care home settings.

**Table 6:** Refinement of the programme theory for future implementation

Primary and secondary programme theories	Refinement to the programme theories for future implementation
(1) If the danceSing care resources are delivered to the care homes, the ACs would consistently deliver the programme to the residents	- The adherence was best when the programme was given two to three times/week out of the four sessions recommended. Therefore, future studies could implement two to three sessions/week
	- For danceSing care staff and researchers, fewer than ten participating care homes would facilitate and speed-up problem solving of any unforeseen barriers regarding the resources, such as tech issues or staffing problems
(2) If the programme is delivered in the care homes, the residents would want to participate regularly	- Participation could be improved if the danceSing care resources are part of the regular care home schedule, with an allocated time slot, playing music at the start of the sessions and briefing and encouraging residents before the start
(3) If the ACs provide the programme consistently, the participants will experience improved psychosocial health markers. Changes in pre-and post-survey data and qualitative interviews would evidence this	- Questionnaires evidenced improvements in several multidimensional health outcomes of participants. Indicators of frailty should be complemented with physical function tests. More objective measures such as actigraphs or endocrine measurements could provide additional information about specific health parameters
(4) If the ACs were given enough organisational support (e.g., resources and time), they would be engaged in this programme. This would establish shared learning and co-production between programme developers, care homes, and researchers	- Wider contexts (e.g., the care home system in Scotland or the COVID restrictions) and organisational contexts (e.g., time and staffing) are out of our control. They are therefore not included in this programme refinement. However, before future implementation of the danceSing care resources takes place, tech issues (e.g., internet access or big screens) and staff issues (e.g., a dedicated AC with back-up) could be factored in
(5) If the group sessions were adequately and consistently used, residents would feel more engaged in group activities, creating a community. This communal feeling would increase self-confidence and quality of life and reduce loneliness. This could potentially inspire future usage of the programme's resources	- Some care home residents who participated had cognitive impairment, influencing the adherence and understanding of measurement tools. Considering cognitive status of care home residents, and exploring how to tailor the intervention to those lacking capacity to consent due to cognitive impairment could make findings more generalisable in a future intervention

## Discussion

The findings of this mixed-methods feasibility study on multidimensional health outcomes of a digital PA intervention using music and movement resources were evaluated through a context-mechanism-outcome (CMO) configuration using a realist approach. This method considered the challenging situation that care homes in Scotland, UK were in during the COVID-19 pandemic, as well as the resources available to the care homes and staff. Consequently, the feasibility of this intervention was established as a component of how the programme would translate into the domain of policy and practice. Discovered delivery challenges were (1) motivation and engagement, (2) cognitive impairment and disabilities of the participants and changes in these, (3) death and hospitalisation of the participants and (4) limited staffing and technology resources to deliver the programme as intended. Regarding the secondary aim of performing limited efficacy testing on multidimensional health markers, ACs and residents commented on improved mood, physical health, job satisfaction and social support, with large effect size values and improvements in the questionnaire scores for anxiety, depression, feelings of loneliness, perceived stress and sleep satisfaction, but no changes in fear of falling, domains of general health and appetite. Due to the feasibility nature of this study and the small sample size, pre- to post-intervention data should be considered preliminary. Overall, these findings tentatively support the recommendation to implement a future RCT, with limitations discussed below. The initial programme theory was therefore refined as discussed below.

### Refinement of the programme theory for future implementation

#### Refinement 1: Future intervention could implement two or three sessions/week

The mean adherence to this 12-week intervention at a dose of 3 + 1 sessions per week was 57%. This is lower than the findings of a previous systematic review about the adherence to supervised technology-based exercise programs for 12 weeks in older adults, where the expected adherence range would fluctuate from 70 to 100% [27]. In this study, adherence was best when the programme was given two to three times/week. This is in line with the recommendations of a Taskforce Report about physical activity and exercise for older adults living in care homes, suggesting a frequency of twice a week, with an interval of at least 48 h between sessions [18]. The proposed refinement of a future implementation of these music and movement resources would be one or two movement sessions and one music session per week.

#### Refinement 2: Encouraging participation in the care homes

Participation and engagement are pertinent issues for developing trials and implementing interventions in practice [47]. Research staff and danceSing care staff tried to engage the ACs to personally invite the care home residents into the programme and emphasise the multiple benefits of training programmes, as these strategies are suggested to be important to maximise participation in older adults [48].

Further, throughout the intervention, the danceSing Care team attempted to create community groups to join the 'danceSing care family'. However, these approaches need to be adjusted to get more appeal from ACs and residents in the future, for example, having more interaction on social media pages. Finally, to maximise adherence to these music and movement resources, special care should be given to making the sessions habitual in the care home, as adherence to physical activity interventions in care homes tends to taper off in the long run [49].

#### Refinement 3: Adding questionnaires of multidimensional health markers complemented with objective measures in a possible future RCT

The intervention effects on multidimensional health markers should be treated as tentative due to the feasibility nature of the study, i.e., the small sample and the lack of a control group. Nevertheless, the programme effects suggest benefits to participants' health and well-being, as evidenced by the large effect size values. A future larger scale randomised-controlled trial would be necessary to confirm this. However, these findings are consistent with the literature on the effects of music and movement resources in older adults, indicating positive changes in mental well-being [15, 19–25]. Physical function tests could usefully complement frailty measurement to aid diagnostic accuracy [42, 50]. Further, during ageing, the endocrine regulation of certain hormones gets challenged; e.g., the activity of the adrenocortical cells that produce the major sex steroid precursor dehydroepiandrosterone (DHEA) and dehydroepiandrosterone sulphate (DHEAS) decreases, often alongside a gradual rise in cortisol (stress hormone) release [51, 52]. Therefore, objective measures of this type could complement health outcomes, such as biomarkers of the hypothalamo-pituitary stress axis (cortisol and DHEAS) [11].

The qualitative analysis showed reported improvements in ACs' and residents' mood and physical health, job satisfaction and social support. Similarly, in previous qualitative studies, older adults in interviews and focus groups have reported that PA interventions have improved their perceptions of their physical capacity and overall physical health [53–55]. Previous observations in older adults also found psychological benefits of engaging in physical activity. These benefits include enjoyment and happiness, which comes not only with engaging in physical activity but also with enjoying the company of others in the group and connecting with people [53–55]. Likewise, previous studies have described that care home staff job satisfaction is associated with high recognition of their contributions to the care home and a sense of belongingness, purpose and responsibility in the care home [56–58]. Thus, these qualitative findings correspond with the belief that providing care and support to residents in the care home setting is challenging but exciting and fulfilling to care home staff [56–58]. Similarly, conducting research in care homes has been reported to be beneficial to care home staff through training and empowerment [59].

#### Refinement 4: Considering dementia in the care home population

Although no data about cognition was formally collected in the current study, it should be noted that it was observed that many of the included participants suffered a significant cognitive decline over the course of the programme. This affected participation and, in this instance, significantly reduced the number of post-intervention assessments possible. One previous study showed that residents who accepted technology-based resources were significantly less cognitively impaired than those who did not [60], adding to the relevance of considering and measuring cognitive status and extending recruitment to those lacking capacity to consent, where possible. In addition, the adherence to an exercise session of an RCT with residents having dementia showed similar adherence rates to this study [61]. The reason for the differences in adherence rates between residents with and without dementia could be that in people without or with only mild cognitive impairment, the instructions and explanations on exercise and its benefits that are usually used to promote PA [62] come across more clearly, possibly increasing adherence. This approach is not feasible in the presence of advanced cognitive impairment as individuals cannot process and remember verbal instructions, highlighting the need for individually tailored activities to functional ability and interest [63]. However, real-time reinforcement and feedback while exercising could improve adherence and provides an opportunity to monitor performance over time [27]. This task could be attributed to the ACs in the care homes in a future RCT. Further, studies including care home residents with dementia generally use the Mini-Mental State Examination (MMSE) [64] or similar to screen for cognitive impairment [61, 65]. Thus, using a cognitive impairment measure could complement the understanding of the effects of dementia on the feasibility and effectiveness of future interventions. This would not be to screen out residents with dementia as those not able to consent to participate in a research study or complete survey measures might still benefit from participating in the intervention programme itself. This would need careful assessment as well as potentially repeated screening to assess changes in cognitive function across the intervention and repeated consent for measurements throughout such an intervention [47]. Further, a fruitful line of future research would be to explore how to tailor the intervention to those lacking capacity with cognitive impairment. A future trial could also extend to recruiting those with cognitive impairment who lack capacity to consent via personal/nominated consultees.

### Strengths and limitations

Realist evaluation approaches have been advocated for evaluating complex care interventions [66, 67]. This approach steers away from a 'one-size-fits-all' problem-solving approach [29]. Indeed, it has some clear strengths. It uses a sound methodology to consider multiple actors at play in complex situations and translates this into the world of policy and practice. However, these approaches are relatively new compared to the processes commonly used in feasibility studies evaluating health care interventions, with a less rigorous traditional methodology. Also, emphasis was placed on contextual factors during a period of some COVID-19 restrictions in the care home system in Scotland. Therefore, the present conclusions may be subject to temporal and situational changes in how programmes are implemented. Nonetheless, care was taken to reach optimal data integration at multiple levels (methods, interpretation and reporting) in the most objective ways possible. Also, stakeholders' feedback (via interviews, focus and advisory groups) was integrated during the data analysis. This acknowledges the importance of the co-production of stakeholders in terms of future programme implementation. Second, the present study is limited by the small sample size, high attrition, and lack of a control group. However, as this was primarily a feasibility study, an adequately powered RCT design was not implemented at this early stage and would form the next step in evaluating this intervention. Finally, it should be acknowledged that due to restricting recruitment to those with the capacity to consent means that we cannot generalise the present findings to that sub-population which is a common sub-group of care home residents. However, we did not preclude inclusion in the intervention group from those lacking capacity, and some individuals who did participate had cognitive impairment, although this influenced adherence and understanding of the measurement tools. Ideally, future research would examine how best to specifically tailor this intervention to the sub-group of individuals with cognitive impairment and lack of capacity to consent and then extend recruitment to those lacking capacity via personal/nominated consultees.

## Conclusion

This realist evaluation of a feasibility study of a digital music and movement intervention in care homes suggests that such an intervention is feasible, at least in individuals with capacity to consent and mild cognitive impairment. The adherence and delivery of the resources are likely to meet greater success when the following features are adopted: (1) implementing two to three sessions per week, (2) encouraging participation by personally inviting the care home residents into the programme or by making the resource a habitual activity and individually-tailoring the activity to the resident's functional/cognitive ability and interest, (3) complementing existing health questionnaires with physical function tests for frailty screening and/or other objective measures of health and well-being, (4) measuring and taking into account cognitive impairment of the participants including exploring ways to tailor the intervention to those lacking capacity to consent.

## Supplementary Information

**Additional file 1: Supplementary Figure 1. **Programme theories and assumptions.

**Additional file 2: Supplementary Figure 2. **Logic model of danceSing care evaluation.

**Additional file 3: Supplementary Table 3. – **danceSing Care resources description.

**Additional file 4: Supplementary Table 4. – **Care home attendance sheet.

**Additional file 5: Supplementary File 5. – **Interview/Focus group Guide danceSing Care evaluationInterview/focusgroup questions.

**Additional file 6: Supplementary Table 6. – **Weekly notes from care homes on delivery challenges.

## Data Availability

The datasets generated and/or analysed during the current study are not publicly available due to still being used for further analysis and PhD thesis work but will be published upon acceptance of the final manuscript and PhD completion associated with these data but are available from the corresponding author on reasonable request.

## Abbreviations

PA: Physical activity
AC: Activity coordinator
AG: Advisory Group
CH: Care home
CMO: Context, mechanism and outcome
RCT: Randomised controlled trial

## References

[1] Office for National Statistics. Changes in the older resident care home population between 2001 and 2011. 2014. Available from: https://www.ons.gov.uk/peoplepopulationandcommunity/birthsdeathsandmarriages/ageing/articles/changesintheolderresidentcarehomepopulationbetween2001and2011/2014-08-01. Accessed Oct 2021.

[2] OECD. Health at a glance. 2021. Available from: https://www.oecd-ilibrary.org/social-issues-migration-health/health-at-a-glance-2021%5C_ae3016b9-en. Accessed Oct 2021.

[3] Gordon AL, Franklin M, Bradshaw L, Logan P, Elliott R, Gladman JRF (2014). Health status of UK care home residents: a cohort study. Age Ageing.

[4] Marengoni A, Angleman S, Melis R, Mangialasche F, Karp A, Garmen A, Fratiglioni L (2011). Aging with multimorbidity: a systematic review of the literature. Ageing Res Rev.

[5] Salive ME (2013). Multimorbidity in older adults. Epidemiol Rev.

[6] Bao J, Chua KC, Prina M, Prince M (2019). Multimorbidity and care dependence in older adults: a longitudinal analysis of findings from the 10/66 study. BMC Public Health.

[7] Alzheimer’s Society. Facts for the media. 2022. Available from: https://www.alzheimers.org.uk/about-us/news-and-media/facts-media. Accessed Oct 2021.

[8] Sackley C, Levin S, Cardoso K, Hoppitt T (2006). Observations of activity levels and social interaction in a residential care setting. Int J Ther Rehabil.

[9] Rodriguez-Larrad A, Arrieta H, Rezola-Pardo C, Esain I, Mendia-Oria P, Irazusta J (2021). Loss of benefits after cessation of exercise interventions in nursing home residents: randomised controlled trial follow-up. Geriatr Nurs (Minneap).

[10] Lazarus NR, Harridge SDR (2018). The inherent human aging process and the facilitating role of exercise. Front Physiol.

[11] De Nys L, Ofosu EF, Ryde GC, Connelly J, Whittaker AC. Physical activity influences cortisol and dehydroepiandrosterone (Sulfate) levels in older adults: a systematic review and meta-analysis. J Aging Phys Act. 2022;1–22. Available from: https://journals.humankinetics.com/view/journals/japa/aop/article-10.1123-japa.2021-0501/article-10.1123-japa.2021-0501.xml. 10.1123/japa.2021-0501 35981715

[12] Sellami M, Bragazzi NL, Slimani M, Hayes L, Jabbour G, De Giorgio A (2019). The effect of exercise on glucoregulatory hormones: a countermeasure to human aging: insights from a comprehensive review of the literature. Int J Environ Res Public Health.

[13] Chou C-H, Hwang C-L, Wu Y-T (2012). Effect of exercise on physical function, daily living activities, and quality of life in the frail older adults: a meta-analysis. Arch Phys Med Rehabil.

[14] Bherer L, Erickson KI, Liu-Ambrose T (2013). A review of the effects of physical activity and exercise on cognitive and brain functions in older adults. J Aging Res.

[15] Windle G, Hughes D, Linck P, Russell I, Woods B (2010). Is exercise effective in promoting mental well-being in older age? A systematic. Rev Aging Ment Health.

[16] Ofosu E, De Nys L, Connelly J, Ryde G, Whittaker AC. Dimensions of physical activity are important in managing anxiety in older adults: a systematic review and meta-analysis (accepted). J Ageing Phys Act. 2022. Available from: 10.1123/japa.2022-0098. 10.1123/japa.2022-0098 36963410

[17] Strawbridge WJ, Deleger S, Roberts RE, Kaplan GA (2002). Physical activity reduces the risk of subsequent depression for older adults. Am J Epidemiol.

[18] de Souto Barreto P, Morley JE, Chodzko-Zajko W, Pitkala K, Weening-Djiksterhuis E, Rodriguez-Mañas L (2016). International Association of Gerontology and Geriatrics – Global Aging Research Network (IAGG-GARN) and the IAGG European region clinical section recommendations on physical activity and exercise for older adults living in long-term care facilities: a tas. J Am Med Dir Assoc.

[19] Arrieta H, Rezola-Pardo C, Zarrazquin I, Echeverria I, Yanguas JJ, Iturburu M, Irazusta J (2018). A multicomponent exercise program improves physical function in long-term nursing home residents: A randomised controlled trial. Exp Gerontol..

[20] Brustio PR, Liubicich ME, Chiabrero M, Rabaglietti E (2018). Dancing in the golden age: a study on physical function, quality of life, and social engagement. Geriatr Nurs (Minneap).

[21] Cordes T, Schoene D, Kemmler W, Wollesen B (2021). Chair-based exercise interventions for nursing home residents: a systematic review. J Am Med Dir Assoc.

[22] Da Silva JL, Agbangla NF, Le Page C, Ghernout W, Andrieu B (2022). Effects of chronic physical exercise or multicomponent exercise programs on the mental health and cognition of older adults living in a nursing home: A systematic review of studies from the past 10 years. Front Psychol.

[23] Guzmán-García A, Hughes JC, James IA, Rochester L (2013). Dancing as a psychosocial intervention in care homes: a systematic review of the literature. Int J Geriatr Psychiatry.

[24] Hwang PW-N, Braun KL (2015). The effectiveness of dance interventions to improve older adults’ health: A systematic literature review. Altern Ther Health Med.

[25] Low LF, Carroll S, Merom D, Baker JR, Kochan N, Moran F (2016). We think you can dance! A pilot randomised controlled trial of dance for nursing home residents with moderate to severe dementia. Complement Ther Med.

[26] Diener J, Rayling S, Bezold J, Krell-Roesch J, Woll A, Wunsch K (2022). Effectiveness and acceptability of e- and m-health interventions to promote physical activity and prevent falls in nursing homes-a systematic review. Front Physiol.

[27] Valenzuela T, Okubo Y, Woodbury A, Lord SR, Delbaere K (2018). Adherence to technology-based exercise programs in older adults: a systematic review. J Geriatr Phys Ther.

[28] Moore GF, Audrey S, Barker M, Bond L, Bonell C, Hardeman W, Baird J (2015). Process evaluation of complex interventions: medical research council guidance. BMJ.

[29] Pawson R, Manzano-Santaella A (2012). A realist diagnostic workshop. Evaluation.

[30] Westhorp G. Realist impact evaluation: an introduction. 2014. Available from: https://odi.org/en/publications/realist-impact-evaluation-an-introduction/. Accessed May 2022.

[31] Wong G, Westhorp G, Manzano A, Greenhalgh J, Jagosh J, Greenhalgh T (2016). RAMESES II reporting standards for realist evaluations. BMC Med.

[32] Eldridge SM, Chan CL, Campbell MJ, Bond CM, Hopewell S, Thabane L (2016). CONSORT 2010 statement: extension to randomised pilot and feasibility trials. BMJ.

[33] Kempen GIJM, Yardley L, Van Haastregt JCM, Zijlstra GAR, Beyer N, Hauer K (2008). The Short FES-I: A shortened version of the falls efficacy scale-international to assess fear of falling. Age Ageing.

[34] Nelson E, Wasson J, Kirk J, Keller A, Clark D, Dietrich A (1987). Assessment of function in routine clinical practice: description of the COOP Chart method and preliminary findings. J Chronic Dis..

[35] EuroQol Group (1990). EuroQol-a new facility for the measurement of health-related quality of life. Health Policy.

[36] Zigmond AS, Snaith RP (1983). The hospital anxiety and depression scale. Acta Psychiatr Scand.

[37] Cohen S, Kamarck T, Mermelstein R. A global measure of perceived stress. J Health Soc Behav. 1983;24:386–96. Available from: http://www.mindgarden.com/products/pss.htm 6668417

[38] Neto F (2014). Psychometric analysis of the short-form UCLA Loneliness Scale (ULS-6) in older adults. Eur J Ageing.

[39] Djukanovic I, Carlsson J, Årestedt K (2017). Is the Hospital Anxiety and Depression Scale (HADS) a valid measure in a general population 65–80 years old? A psychometric evaluation study. Health Qual Life Outcomes.

[40] Ohayon MM, Paskow M, Roach A, Filer C, Sunshine Hillygus D, Chen MC (2019). The national sleep foundation’s sleep satisfaction tool. J Natl Sleep Found.

[41] Wilson M-MG, Thomas DR, Rubenstein LZ, Chibnall JT, Anderson S, Baxi A, et al. Appetite assessment: simple appetite questionnaire predicts weightloss in community-dwelling adults and nursing home residents. Am J Clin Nutr. 2005;82(5):1074–81. Available from:https://academic.oup.com/ajcn/article/82/5/1074/4607521 10.1093/ajcn/82.5.1074 16280441

[42] Fried LP, Tangen CM, Walston J, Newman AB, Hirsch C, Gottdiener J, et al. Frailty in older adults: evidence for a phenotype. J Gerontol Med Sci. 2001;56(3):146–56. Available from:https://academic.oup.com/biomedgerontology/article/56/3/M146/545770 10.1093/gerona/56.3.m146 11253156

[43] Treacy D, Hassett L (2018). The short physical performance battery. J Physiother.

[44] Podsiadlo D, Richardson S (1991). The timed “Up & Go”: a test of basic functional mobility for frail elderly persons. J Am Geriatr Soc.

[45] Manzano A (2016). The craft of interviewing in realist evaluation. Evaluation.

[46] Cohen J (1988). Statistical Power Analysis for the Behavioral Sciences.

[47] Yardley L, Donovan-Hall M, Francis K, Todd C (2007). Attitudes and beliefs that predict older people’s intention to undertake strength and balance training. J Gerontol B Psychol Sci Soc Sci.

[48] Yardley L, Bishop FL, Beyer N, Hauer K, Kempen GIJM, Piot-Ziegler C (2006). Older people’s views of falls-prevention interventions in six European countries. Gerontologist.

[49] Nyman SR, Victor CR (2011). Older people’s recruitment, sustained participation, and adherence to falls prevention interventions in institutional settings: a supplement to the Cochrane systematic review. Age Ageing.

[50] Clegg A, Young J, Iliffe S, Rikkert MO, Rockwood K (2013). Frailty in elderly people. Lancet.

[51] Heaney JLJ, Carroll D, Phillips AC. Physical activity, life events stress, cortisol, and DHEA : preliminary findings that physical activity may buffer against the negative effects of stress. J Ageing Phys Act. 2014;22:465–73. 10.1123/japa.2012-0082 24084142

[52] Chehab O, Ouertani M, Chaieb K, Haouala F, Mahdouani K (2007). Hormonal status of cortisol and dehydroepiandrosterone sulfate in an elderly Tunisian population. C R Biol.

[53] Lindsay-Smith G, Eime R, O’Sullivan G, Harvey J, van Uffelen JG (2019). A mixed-methods case study exploring the impact of participation in community activity groups for older adults on physical activity, health and wellbeing. BMC Geriatr.

[54] Maula A, LaFond N, Orton E, Iliffe S, Audsley S, Vedhara K (2019). Use it or lose it: a qualitative study of the maintenance of physical activity in older adults. BMC Geriatr.

[55] Swales B, Ryde GC, Whittaker AC (2022). A randomized controlled feasibility trial evaluating a resistance training intervention with frail older adults in residential care: the keeping active in residential elderly trial. J Aging Phys Act.

[56] Foà C, Guarnieri MC, Bastoni G, Benini B, Giunti OM, Mazzotti M (2020). Job satisfaction, work engagement and stress/burnout of elderly care staff: A qualitative research. Acta Bio Medica Atenei Parm.

[57] Hirakawa Y, Chiang C, Uemura MY, Aoyama A (2019). Job satisfaction among Japanese home-visit care workers. Home Health Care Manag Pract.

[58] Moyle W, Skinner J, Rowe G, Gork C (2003). Views of job satisfaction and dissatisfaction in Australian long-term care. J Clin Nurs.

[59] Jenkins C, Smythe A, Galant-Miecznikowska M, Bentham P, Oyebode J (2016). Overcoming challenges of conducting research in nursing homes. Nurs Older People.

[60] Ulbrecht G, Wagner D, Gräßel E (2012). Exergames and their acceptance among nursing home residents. Act Adapt Aging.

[61] Henskens M, Nauta IM, Drost KT, Scherder EJ (2018). The effects of movement stimulation on activities of daily living performance and quality of life in nursing home residents with dementia: a randomised controlled trial. Clin Interv Aging.

[62] Spittaels H, De Bourdeaudhuij I, Vandelanotte C (2007). Evaluation of a website-delivered computer-tailored intervention for increasing physical activity in the general population. Prev Med (Baltim).

[63] Hill NL, Kolanowski A, Kürüm E (2010). Agreeableness and activity engagement in nursing home residents with dementia. J Gerontol Nurs.

[64] Folstein MF, Folstein SE, McHugh PR (1975). Mini-mental state: A practical method for grading the cognitive state of patients for the clinician. J Psychiatr Res.

[65] Thurm F, Scharpf A, Liebermann N, Kolassa S, Elbert T, Lüchtenberg D, Kolassa I-T (2011). Improvement of cognitive function after physical movement training in institutionalized very frail older adults with dementia. GeroPsych (Bern).

[66] Byng R, Norman I, Redfern S (2005). Using realistic evaluation to evaluate a practice-level intervention to improve primary healthcare for patients with long-term mental illness. Evaluation.

[67] Salter KL, Kothari A (2014). Using realist evaluation to open the black box of knowledge translation: a state-of-the-art review. Implement Sci.

