# Supplementary figures and images for: A realist evaluation of the feasibility of a randomised controlled trial of a digital music and movement intervention for older people living in care homes
Source: BMC Geriatr. 2023 Mar 6;23:125. doi: 10.1186/s12877-023-03794-5 (PMC9987360; doi:10.1186/s12877-023-03794-5)

**Supplementary Figure 1: Programme theories and assumptions**


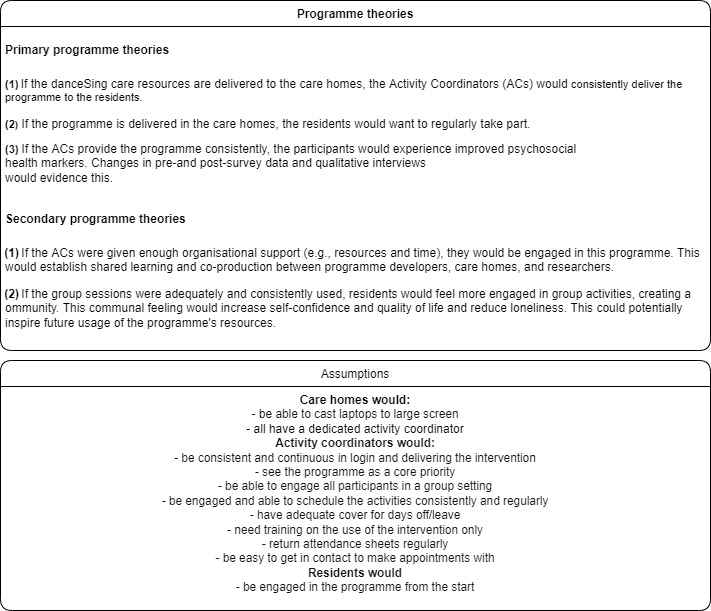

Supplement: Supplementary file 1 — Additional file 1: Supplementary Figure 1. Programme theories and assumptions. [file 12877_2023_3794_MOESM1_ESM.docx]

**Supplementary Figure 2: Logic model of danceSing care evaluation**


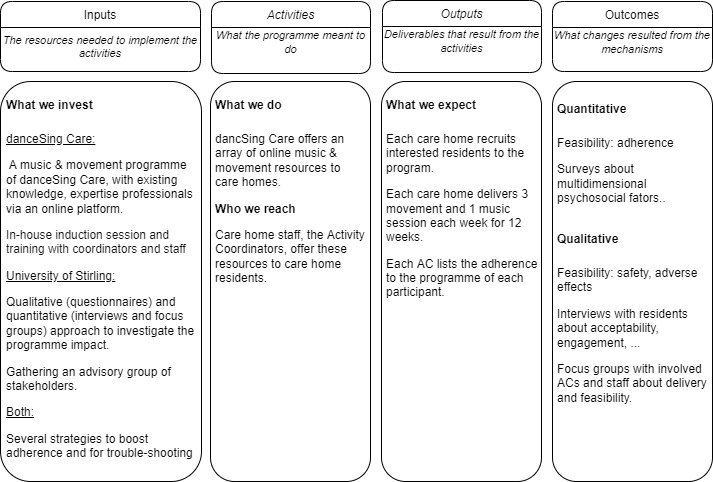

Supplement: Supplementary file 2 — Additional file 2: Supplementary Figure 2. Logic model of danceSing care evaluation. [file 12877_2023_3794_MOESM2_ESM.docx]
