# Supplementary material for: A realist evaluation of the feasibility of a randomised controlled trial of a digital music and movement intervention for older people living in care homes
Source: BMC Geriatr. 2023 Mar 6;23:125. doi: 10.1186/s12877-023-03794-5 (PMC9987360; doi:10.1186/s12877-023-03794-5)
Supplement: Supplementary file 3 — Additional file 3: Supplementary Table 3. – danceSing Care resources description. [file 12877_2023_3794_MOESM3_ESM.docx]

**Supplementary Table 3 – danceSing Care resources description**

**MUSIC AND SINGING PROGRAMME - session list**

Our Music and Singing Programme is led by highly experienced danceSing Music Leader, Karol Fitzpatrick on piano.  Upbeat and calming sessions, our chosen songs will stimulate memory and provide a range of tempi and mood. Our shorter session bites can be practised regularly and will help to improve breathing, posture, vocal control, concentration, and co-ordination.

| **Session** | **Duration** | **Overview** | **Dementia Friendly** | **Key Tags** |
| --- | --- | --- | --- | --- |
| **GET STARTED SERIES** - Sing along or listen to our bespoke musical arrangements to improve overall well-being. | | | | |
| 1 | 25-minutes | **Sing along** to ‘Yes Sir I Can Boogie’ and sit back and enjoy to ‘Moon River’. | Mild  Moderate | Music, Singing, Calm, Relax, Upbeat |
| 2 | 22-minutes | **Sing along** to ‘That’s The Way I Like It’ and sit back and enjoy ‘Somewhere Over The Rainbow’ and ‘Raindrops Keep Falling On My Head’. | Mild  Moderate | Music, Singing, Calm, Relax, Upbeat |
| 3 | 26-minutes | **Sing along** to ‘The Lion Sleeps Tonight’ and sit back and enjoy ‘Fields Of Gold’ and ‘What A Wonderful World’. | Mild  Moderate | Music, Singing, Calm, Relax, Upbeat |
| 4 | 7-minutes | **Sing along** or sit back and enjoy ‘Scarborough Fair’ and ‘Peace In My Soul’. | All Stages | Music, Singing, Calm, Relax |
| 5 | 22-minutes | **Sing Along** to or sit back and enjoy “Amazing Grace” and “Somewhere Over The Rainbow” with accompanying grand piano and flute. | All Stages | Music, Singing, Calm, Relax |
| **IRISH SERIES** - Well known Irish songs to stimulate memory and offer a range of tempi and mood. | | | | |
| 1 | 19-minutes | **Sing along** to ‘I’ll Tell Me Ma’ and ‘The Wild Rover’. | All Stages | Music, Singing, Upbeat, Energise |
| 2 | 21-minutes | **Sing along** to ‘Molly Malone’ and ‘Danny Boy’. | All Stages | Music, Singing, Calm, Relax |
| **FESTIVE SERIES -** Sing along or listen to our favourite selection of magical Christmas Carols. | | | | |
| 1 | 20-minutes | **Sing along** to ‘Winter Wonderland’ | All Stages | Music, Singing, Upbeat, Energise |
| 2 | 25-minutes | **Sing along** to ‘White Christmas’ | All Stages | Music, Singing, Calm, Relax |
| **BITESIZE SESSIONS -** Selection of shorter bitesize sessions including Feel-good, Ballet & Irish (approx. 5-mins long). | | | | |
| 1 | 5-minutes | **GENERAL Sing along bite** Voice Strengthening Exercise | Mild  Moderate | Music, Singing, Calm, Relax |
| 2 | 5-minutes | **IRISH Sing along bite ‘**Molly Malone’ | All Stages | Music, Singing, Calm, Relax |
| 3 | 6-minutes | **IRISH Sing along bite ‘**The Wild Rover’ | All Stages | Music, Singing, Upbeat, Energise |
| 4 | 6-minutes | **IRISH Sing along bite** ‘Danny Boy’ | All Stages | Music, Singing, Calm, Relax |
| 5 | 5-minutes | **FESTIVE Sing along bite** ‘White Christmas’ | Mild  Moderate | Music, Singing, Upbeat, Energise |
| 6 | 5-minutes | **FESTIVE Sing along bite** ‘We Wish You A Merry Christmas’ | Mild  Moderate | Music, Singing, Upbeat, Energise |

**MOVEMENT AND FITNESS PROGRAMME - session list**

Our Movement and Fitness Programme is led by highly experienced danceSing Founder Natalie Garry. Inspiring and motivational, our sessions will help prevent falls, improve posture, boost mood and circulation, increase strength and flexibility to help with everyday activities and promote independent living.

| **Session** | **Duration** | **Overview** | **Dementia Friendly** | **Key Tags** |
| --- | --- | --- | --- | --- |
| **GET STARTED SERIES** - Have fun moving and grooving to boost your overall well-being. | | | | |
| 1 | 18-minutes | **Chair Fitness** focusing on improving posture, strength, and flexibility, boosting cardiovascular health, and mobilising joints. | Mild  Moderate | Chair, Dance, Calm, Relax, Upbeat, Circulation Falls, Energise |
| 2 | 19-minutes | **Chair Fitness** focusing on improving posture, strength, and flexibility, boosting cardiovascular health, and mobilising joints. | Mild  Moderate | Chair, Dance, Calm, Relax, Upbeat, Circulation Falls, Energise |
| 3 | 18-minutes | **Standing Fitness** focusing on improving posture, strength, and flexibility, boosting cardiovascular health, and mobilising joints. | Mild  Moderate | Standing, Dance, Calm, Relax, Upbeat, Circulation Falls, Energise |
| 4 | 8-minutes | **Chair Fitness Circulation Boost** focusing on boosting cardiovascular health, mobilising joints, and improving posture, strength, and flexibility. Recommended daily. | Mild  Moderate | Chair, Dance, Calm, Relax, Upbeat, Circulation Falls, Energise |
| 5 | 21-minutes | **Chair Fitness** focusing on boosting circulation, cardiovascular health, mobilising joints, and improving posture, strength and flexible. | Mild  Moderate | Chair, Dance, Calm, Relax, Upbeat, Circulation, Falls, Energise |
| 6 | 11-minutes | **Chair Fitness Circulation Boost** focusing on cardiovascular health, mobilising joints, and improving posture, strength, and flexibility. Recommended daily. | All Stages | Chair, Dance, Calm, Relax, Circulation, Falls, Energise |
| 7 | 11-minutes | **Chair Fitness** focusing on easing out the muscles and improving posture and flexibility. Recommended daily. | All Stages | Chair, Dance, Calm, Relax, Circulation, Falls |
| 8 | 12-minutes | **Chair Fitness** focusing on moving gently, mobilising the joints, and improving posture and flexibility. Recommended daily. | All Stages | Chair, Dance, Calm, Relax, Circulation, Falls |
| **FEEL-GOOD SERIES** - Motivational feel-good sessions created to help enable independent living. | | | | |
| 1 | 21-minutes | **Chair Fitness** focussing on improving posture, boosting circulation, falls prevention, and increased strength for everyday activities. | Mild  Moderate | Chair, Fitness**,** Upbeat, Circulation Falls, Energise |
| 2 | 21-minutes | **Standing Fitness** for people steady on their feet. Focusing on maintaining and improving movement quality and agility while working on falls prevention, increasing strength and flexibility for everyday activities. | Mild  Moderate | Standing, Fitness, Upbeat, Circulation Falls, Energise |
| 3 | 12-minutes | **Gentle Chair Fitness** slower paced focussing on gentle movements to mobilise the body. | All Stages | Chair, Fitness, Upbeat, Circulation Falls, Energise |
| **BALLET SERIES** - Joyful and uplifting inspired by classical ballet to improve strength for everyday activities. | | | | |
| 1 | 22-minutes | **Chair Fitness** focussing on improving posture, boosting circulation, falls prevention, increased strength for everyday activities. | Mild  Moderate | Chair, Fitness, Upbeat, Circulation Falls, Energise |
| 2 | 19-minutes | **Standing Fitness** focussing on maintaining and improving movement quality and agility while working on falls prevention, increasing strength and flexibility for everyday activities. | Mild  Moderate | Standing, Fitness, Upbeat, Circulation Falls, Energise |
| 3 | 13-minutes | **Chair Relaxation** focussing on breath work and gentle movement to mobilise the body leaving you feeling calm and relaxed. | All Stages | Chair, Fitness, Calm, Relax, Circulation, Falls |
| **FESTIVE SERIES** - Festive fun to improve posture and circulation, prevent falls, increase strength and flexibility. | | | | |
| 1 | 18-minutes | **Chair Fitness** focusing on improving posture, boosting circulation, falls prevention, and increased strength and flexibility for everyday activities. | All Stages | Chair, Fitness, Upbeat, Circulation Falls, Energise |
| **BITESIZE SESSIONS -** Selection of shorter bitesize sessions including Feel-good, Ballet & Irish (max. 5-mins long). | | | | |
| 1 | 4-minutes | **FEEL-GOOD Chair Bite** ‘Don’t Worry Be Happy’ | Mild  Moderate | Chair, Fitness, Upbeat, Circulation Falls, Energise |
| 2 | 4-minutes | **FEEL-GOOD Chair Bite** ‘Fragile’ | All Stages | Chair, Fitness, Calm, Relax, Circulation, Falls |
| 3 | 3-minutes | **FEEL-GOOD Standing Bite** ‘The Lion Sleeps Tonight’ | All Stages | Standing, Fitness, Upbeat, Circulation Falls, Energise |
| 4 | 4-minutes | **BALLET Chair Bite** Mobility Circulation | All Stages | Chair, Fitness, Calm, Relax, Circulation, Falls |
| 5 | 7-minutes | **BALLET Standing Bite** Mobility Circulation | All Stages | Standing, Fitness, Upbeat, Circulation Falls, Energise |
| 6 | 3-minutes | **BALLET Chair Bite** Posture and Breath Work | All Stages | Chair, Fitness, Upbeat, Circulation Falls, Energise |

**MEMORY LANE RADIO - show list**

Take a trip down Memory Lane, unlocking memories through the magic of music and movement.

| **Session** | **Duration** | **Overview** | **Dementia Friendly** | **Key Tags** |
| --- | --- | --- | --- | --- |
| 1 | 60-minutes | Let danceSing transport you through the decades (50s, 60s, 70s, & 80s) with a variety of **CLASSICAL** and **POPULAR** music. | All Stages | Radio, Music, Calm, Relax, Upbeat |
| 2 | 30-minutes | Let danceSing take you on a musical trip down memory lane with their handpicked selection of **1950s** classics. | All Stages | Radio, Music, Calm, Relax, Upbeat |
| 2 | 30-minutes | Let danceSing take you on a musical trip down memory lane with their handpicked selection of **1960s** classics. | All Stages | Radio, Music, Calm, Relax, Upbeat |
| 3 | 30-minutes | Let danceSing take you on a musical trip down memory lane with their handpicked selection of **JAZZY** **FESTIVE** classics. | All Stages | Radio, Music, Calm, Relax, Upbeat |
| 4 | 30-minutes | Let danceSing take you on a musical trip down memory lane with their handpicked selection of **JAZZY** **FESTIVE** classics. | All Stages | Radio, Music, Calm, Relax, Upbeat |
| 5 | 30-minutes | Let danceSing take you on a musical trip down memory lane with their handpicked selection of **TRADITIONAL** **FESTIVE** classics. | All Stages | Radio, Music, Calm, Relax, Upbeat |

**MUSICAL CONCERTS - show list**

Sit back, relax, and enjoy our magical danceSing Care musical concert.

| **Show** | **Duration** | **Overview** | **Dementia Friendly** | **Key Tags** |
| --- | --- | --- | --- | --- |
| 1 | 20-minutes | **POPULAR** music medley performance of grand piano and flute covering a variety of popular uplifting music. | All Stages | Concert, Music, Singing, Calm, Relax, Upbeat |
| 2 | 18-minutes | **IRISH** music medley performance of grand piano and flute covering a variety of popular uplifting music. | All Stages | Concert, Music, Singing, Calm, Relax, Upbeat |
| 3 | 12-minutes | **FESTIVE** music medley performance of grand piano and flute covering a variety of popular uplifting music. | All Stages | Concert, Music, Singing, Calm, Relax, Upbeat |
