# Supplementary material for: A realist evaluation of the feasibility of a randomised controlled trial of a digital music and movement intervention for older people living in care homes
Source: BMC Geriatr. 2023 Mar 6;23:125. doi: 10.1186/s12877-023-03794-5 (PMC9987360; doi:10.1186/s12877-023-03794-5)
Supplement: Supplementary file 4 — Additional file 4: Supplementary Table 4. – Care home attendance sheet. [file 12877_2023_3794_MOESM4_ESM.docx]

**Supplementary Table 4 – Care home attendance sheet**

Care home name: _______________

**danceSing Care attendance:** please tick the corresponding box each time a resident participating in the danceSing care Evaluation (i.e. they did the baseline survey) attended a **movement class** in the program and a circle for the **music** **only** class.

Please scan and send to us each Friday so we can keep a cumulative attendance record.

| **W1** | **W 2** | **W3** | **W4** | **W5** | **W6** | **W7** | **W8** | **W9** | **W10** | **W11** | **W12** |
| --- | --- | --- | --- | --- | --- | --- | --- | --- | --- | --- | --- |
|   |   |   |   |   |   |   |   |   |   |   |   |
|  |  |  |  |  |  |  |  |  |  |  |  |
|   |   |   |   |   |   |   |   |   |   |   |   |
|  |  |  |  |  |  |  |  |  |  |  |  |
|   |   |   |   |   |   |   |   |   |   |   |   |
|  |  |  |  |  |  |  |  |  |  |  |  |
|   |   |   |   |   |   |   |   |   |   |   |   |
|  |  |  |  |  |  |  |  |  |  |  |  |
|   |   |   |   |   |   |   |   |   |   |   |   |
|  |  |  |  |  |  |  |  |  |  |  |  |

**NAME**
