# Supplementary material for: A realist evaluation of the feasibility of a randomised controlled trial of a digital music and movement intervention for older people living in care homes
Source: BMC Geriatr. 2023 Mar 6;23:125. doi: 10.1186/s12877-023-03794-5 (PMC9987360; doi:10.1186/s12877-023-03794-5)
Supplement: Supplementary file 5 — Additional file 5: Supplementary File 5. – Interview/Focus group Guide danceSing Care evaluationInterview/focusgroup questions. [file 12877_2023_3794_MOESM5_ESM.docx]

**Supplementary File 5 – Interview/Focus group Guide**

**danceSing Care evaluation**

**Interview/focus group questions**

**Semi-structured interview with residents**

**General overview of participation/adherence**

1. What did you think about the danceSing Care online activities?
2. Did you manage to take part in the 3 movement and 1 music sessions a week? If not, why?
3. Can you tell me about your typical music and movement session?
4. What do you think about the length of the sessions? (Probe- would you say sessions were too long or too short)
5. Did you complete most of the sessions or had to stop at any point?
6. What did you like about the danceSing care activities?

**Follow up questions**

Did you prefer the movement or music activities? (depending on Q6 answer)

Is there anything you think was missing from the danceSing care sessions?

1. Was there anything you did not like?
2. What made you continue/stop to take part in the music and movement activities?

**Residents’ participation/benefits**

1. Do you think the activities have improved your life in any way?

In what ways?

1. Would you like to continue to take part in the danceSing care activities? Why/why not?

**Social support**

1. Do you feel part of the danceSing Care family?
2. Has engaging in the music and movement activities brought you closer to other residents and staff?

**Semi-structured focus group with staff**

Go round for everyone to introduce themselves, role and the care home they work with.

**General overview of participation/adherence**

1. Can you tell me what your involvement in this danceSing care program has been ? (considering we might have other care home staff joining the FG)
2. Did you manage engaging residents in the 3+1 sessions a week? If not, why not?- mechanism
3. Did residents complete sessions after stating? If not why?
4. Do you think the residents enjoyed taking part in the danceSing care activities?

Can you give some examples

1. Did you enjoy taking part in the danceSing care activities?- outcome
2. Did you prefer the music/movement or the singing activities?

**Residents’ and staff participation/benefits**

1. What type of resident would you say came to a typical danceSing care session?
2. Did some residents use the danceSing care activities more than others? If so, why?
3. Did the class size grow or reduce? If so, why?
4. What do you think were the benefits of the danceSing care activities for residents?
5. What were any benefits of the danceSing care activities for you?
6. Would you like to carry on using the danceSing care activities with residents? Why/why not?

**Delivery of danceSing care program**

1. How did you find delivering the program?
2. What would you say helped with delivering the program?
3. Would you say delivering the program came with some challenges?

If so, in what ways?

1. If you could change something about this programme to make it work effectively in the care home, what would you change and why?

**Social support**

1. Do you feel part of danceSing care family/champions?
2. Would you say delivering the danceSing care activities has improved social connections between residents and staff?
