# Supplementary material for: A realist evaluation of the feasibility of a randomised controlled trial of a digital music and movement intervention for older people living in care homes
Source: BMC Geriatr. 2023 Mar 6;23:125. doi: 10.1186/s12877-023-03794-5 (PMC9987360; doi:10.1186/s12877-023-03794-5)
Supplement: Supplementary file 6 — Additional file 6: Supplementary Table 6. – Weekly notes from care homes on delivery challenges. [file 12877_2023_3794_MOESM6_ESM.docx]

**Supplementary Table 6 – Weekly notes from care homes on delivery challenges**

| Weekly reports from care home staff through phone calls and emails | | | | | | |
| --- | --- | --- | --- | --- | --- | --- |
| Week 1 | CH1 struggled to get residents to participate | CH2 recorded two drop outs. 1 relocation and 1 withdrawal | CH4- AC went on holiday and ran only one session | CH5 and CH6 had no assigned ACs |  |  |
| Week 2 | CH7 reported all residents have dementia and attention span is short making full participation difficult | CH3- COVID outbreak in the care home and Ac is on holiday. |  |  |  |  |
| Week 3 | CH3- COVID outbreak in home and one residents passed away | CH7-attendance forms not received | CH2- AC resigned and no new reassigned. | Less attention of ACs to weekly mails and reminders | Poor engagement on the social media platform for ACs |  |
| Week 4 | CH5 recruited a new AC who is yet to start | CH3- COVID outbreak | CH8- COVID outbreak. Residents are isolating and not engaging in any social activities |  |  |  |
| Week 5 | CH9- AC is off sick. No sessions were run | CH8- COVID outbreak. Residents are isolating and not engaging in any social activities | CH3- one resident hospitalised | CH7- AC reported intervention has been delivered for only 2 weeks. AC was reminded of attendance forms | CH1- AC was on holidays so sessions were not delivered |  |
| Week 6 | CH8- COVID outbreak. Residents are isolating and not engaging in any social activities | CH1- AC was on holidays so sessions were not delivered | CH2- New AC starting next week. | CH6- New Ac starting in 2 weeks. | CH9-AC off sick with COVID | CH4- AC on holidays and delivery of sessions taken over by carer. |
| Week 7 | CH4- AC on holidays and delivery of sessions taken over by carer. | CH10- One resident hospitalised | CH7, CH5, CH6 have not provided any attendance data- no sessions delivered |  |  |  |
| Week 8 | CH5- Residents struggle with the movement sessions |  |  |  |  |  |
| Week 9 | CH7- No response to follow up calls | CH2- New AC was off due to family issues. Care staff were unable to give any updates |  |  |  |  |
| Week 10 | CH7- AC is off on holidays | CH10: AC unable to join Facebook page. | CH2- Care home staff reported sessions have stopped due to staff shortage. | CH8 - no sessions delivered due to COVID. AC reported sessions were difficult to follow (too fast) for residents. | CH1- attendance is better now since faulty display screen has been repaired |  |
| Week 11 and 12 | Final phone call follow ups and email reminders for attendance sheets made to all care homes | CH5, CH6 and CH7 did not engage in the intervention |  |  |  |  |

*Note. CH: Care home, AC: Activity Coordinator*
